# Case of Sudden Death

**Published:** 1869-01

**Authors:** A. M. Gray

**Affiliations:** Chicago


ARTICLE IV.

### CASE OF SUDDEN DEATH.

By A. M. GRAY, M.D., Chicago.

On Saturday, Nov. 21st, 1868, at noon, I was summoned, in great haste, to see a man who (the messenger said) had fallen down on the street near by, in a fit.

Accompanying the messenger, I found a Bohemian wood-sawyer, 38 years of age, lying in a store, writhing with pain. He could understand no English, and no interpreter was at hand, but by signs he gave me to understand that the difficulty was at the top of the sternum. He made no attempt at talking, nor did he even moan, but he was with great difficulty kept quiet. The extremities were *very* cold, although he was warmly clad. No pulse whatever could I discover. Countenance haggard and anxious; lips deeply congested; brow covered with cold, clammy sweat; inspiration difficult; expiration easy; by auscultation, little or no movement of the heart could be discovered. Diagnosis, "angina pectoris." I immediately administered a drachm of chloroform internally, with the effect of relieving the paroxysm for perhaps five minutes, when another paroxysm came on: he now indicated that the pain had shifted to the base of the sternum. In another moment he exhibited signs of great pain in the bladder, and at the occiput. By this time a hot paste of mustard and capsicum was prepared, and was applied *liberally* to the chest and stomach. Another drachm of chloroform was administered, followed by relief. He showed signs of sinking, and I administered quinine sulph., grs. x.; pulv. capsici, grs. v. But our efforts were of no avail. In twenty minutes from the time I was called he was dead. Consciousness was maintained to within five minutes of his death.

An autopsy was denied.

From the wife I learned that he had been in this country about a year, that he had never known a sick day, had never complained of any pain, except a little soreness in the chest for the past few weeks; that he was regular in his habits, temperate, appetite good. I should judge him to have been about five feet ten inches in height, weight about 175 lbs., though not fleshy; countenance slightly anæmic. From the symptoms and appearances of the patient, I am inclined to the opinion that fatty degeneration of the heart, or dilatation of its walls, existed.

